# The Prevalence of Depressive and Anxiety Symptoms Among Healthcare Workers of East Avenue Medical Center (EAMC) During the COVID-19 Pandemic

**DOI:** 10.1192/j.eurpsy.2022.1377

**Published:** 2022-09-01

**Authors:** M.Y.E. Pereyra-Borlongan

**Affiliations:** EAST AVENUE MEDICAL CENTER, Internal Medicine, QUEZON CITY, Philippines

**Keywords:** Anxiety, Covid-19, Health Care Worker, Depression

## Abstract

**Introduction:**

COVID-19, caused by the most recently discovered corona virus SARS-CoV-2 (Severe Acute Respiratory Syndrome Corona Virus 2), has already reached pandemic scale worldwide. And it is expected for health care workers to feel stressed and worried during these times due to their exposure to infected individuals.

**Objectives:**

This study aims to identify the relationship between the demographic profile and health-related traits of EAMC employees, and their perceived stressors that aggravate psychologic distress experienced during the COVID-19 pandemic.

**Methods:**

In this cross-sectional study of 390 health care workers, we use two brief mental health screening tools, namely Patient Health Questionnaire-9 (PHQ-9), a validated tool for depression, and Generalized Anxiety Disorder Scale-7 (GAD-7) a validated tool for anxiety.

**Results:**

Factors such as marital status, occupation and employment status appear to have association with the development of depressive and anxiety symptoms. Perceived stressors during the COVID-19 pandemic include long working hours, inconsistent administrative policies, exposure to Covid-19 positive individuals, inadequate time off from work and increasing number of patients but without commensurate additional work force. For our sample population, 31.8% did not have depressive symptoms, 35.6% had mild, 21% moderate, 9.7% moderately severe and 1.8% had severe depressive symptoms. While for anxiety, 57.7% had no to minimal symptoms, 26.9% mild, 9.7% moderate and 5.6% had severe anxiety symptoms.

**Conclusions:**

It is therefore concluded that the Covid-19 pandemic indeed caused significant anxiety and depressive symptoms among health care workers. Measures to increase the work force should be put in place to decrease work burden and employee fatigue.

**Disclosure:**

No significant relationships.

